# Supplementary material for: Pentastiridius leporinus (Linnaeus, 1761) as a Vector of Phloem-Restricted Pathogens on Potatoes: ‘Candidatus Arsenophonus Phytopathogenicus’ and ‘Candidatus Phytoplasma Solani’
Source: Insects. 2024 Mar 13;15(3):189. doi: 10.3390/insects15030189 (PMC10971734; doi:10.3390/insects15030189)
Supplement: Supplementary file 1 [file insects-15-00189-s001.zip › insects-2909947-supplementary.pdf]

Supplementary material

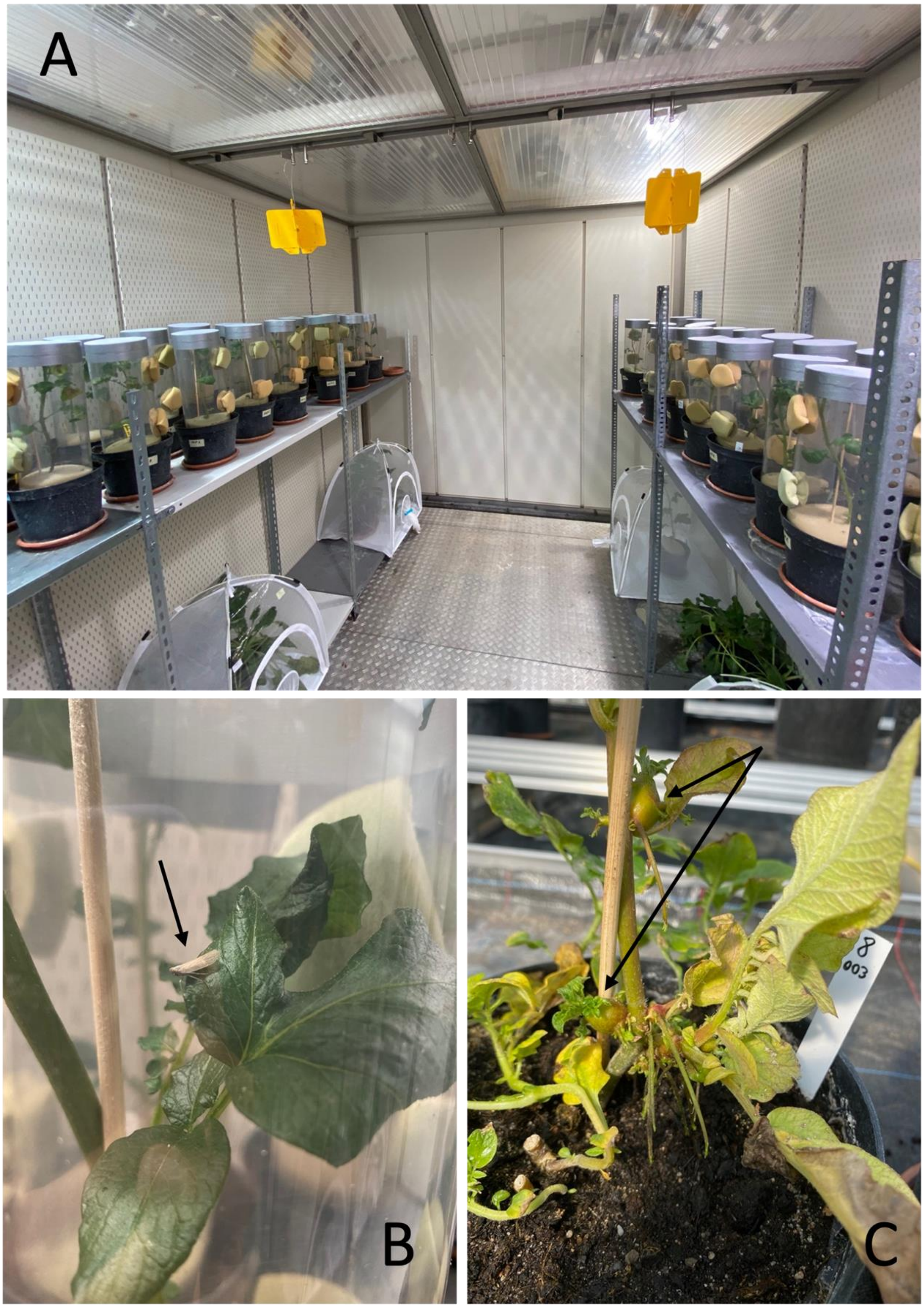

Figure S1. Photos of the experimental set-up of the transmission trial. A: Cylinders of the transmission experiment in the climate chamber; B: A female of *Pentastiridius leporinus* inside a transmission cylinder; C: Arrows are pointing to aerial tubers of an infected transmission plant.

Table S1. Results of the transmission experiment in more detail. The number of CAp- and CPs-positive insects and potato tubers was determined by real-time PCR analysis.

Variety	Plant number					Total
	#1	#2	#3	#4	#5	(infection rate)
**Lilly**						
Insects tested (total)	9	8	10	8	10	45
CAp-positive	9	8	10	8	10	45 (100%)
CPs-positive	1	0	0	0	0	1 (2.2%)
Insects not tested	1	2	0	2	0	5
Tubers tested (total)	3	3	3	3	3	15
CAp-positive	2	0	1	0	1	4 (26.7%)
CPs-positive	0	0	0	0	0	0
**Merle**						
Insects tested (total)	9	10	7	7	9	42
CAp-positive	9	10	7	7	9	42 (100%)
CPs-positive	3	1	0	1	0	5 (11.9%)
Insects not tested	1	0	3	3	1	8
Tubers tested (total)	3	3	3	3	3	15
CAp-positive	0	0	0	0	0	0
CPs-positive	1	0	1	1	0	3 (20%)
**Belana**						
Insects tested (total)	9	9	10	10	9	47
CAp-positive	9	9	10	10	9	47 (100%)
CPs-positive	0	2	0	0	0	2 (4.2%)
Insects not tested	1	1	0	0	1	3
Tubers tested (total)	3	3	3	3	3	15
CAp-positive	1	1	0	2	0	4 (26.7%)
CPS-positive	0	0	0	0	0	0
**Juventa**						
Insects tested (total)	9	9	10	10	3	41
CAp-positive	9	9	10	10	3	41 (100%)
CPs-positive	0	0	0	0	0	0
Insects not tested	1	1	0	0	7	9
Tubers tested (total)	3	2	3	3	3	14
CAp-positive	0	1	3	0	0	4 (28.6%)
CPs-positive	0	0	0	0	0	0
**Gala**						
Insects tested (total)	7	11	10	10	9	47
CAp-positive	7	11	10	10	9	47 (100%)
CPs-positive	0	0	6	0	0	6 (12.8%)
Insects not tested	3	0	0	0	1	4
Tubers tested (total)	3	3	3	3	3	15
CAp-positive	0	2	1	3	3	9 (60%)
CPs-positive	0	0	0	0	0	0
